# Registerdaten zur zementierten Endoprothetik: Belegen sie den Trend zur zementfreien Versorgung?

**DOI:** 10.1007/s00132-023-04451-w

**Published:** 2023-10-27

**Authors:** Martina Humez, Katharina Kötter, Ralf Skripitz, Klaus-Dieter Kühn

**Affiliations:** 1 Heraeus Medical GmbH, Philipp-Reis-Str. 8/13, 61273 Wehrheim, Deutschland; 2 Zentrum für Endoprothetik, Fußchirurgie, Kinder- und Allgemeine Orthopädie, Roland-Klinik Bremen, Bremen, Deutschland; 3 Department of Orthopaedics and Trauma, Medizinische Universität Graz, Graz, Österreich

**Keywords:** Antibiotika, Knochenzement, Endoprothesen, Periprothetische Fraktur, Revisionschirurgie, Antibiotics, Bone cement, Endoprosthesis, Periprosthetic fractures, Revision surgery

## Abstract

**Hintergrund:**

In der Endoprothetik gibt es Hüft- und Knieimplantate, die zementfrei, „press-fit“ oder mit Knochenzement verankert werden. Real-World-Evidenz aus Endoprothesenregistern sowie Studien bieten eine breite Datenbasis zur Diskussion von zementierten im Vergleich zu zementfreien Endoprothesen.

**Fragestellung:**

Wie lautet die Empfehlung zur zementierten oder zementfreien Verankerung von Hüft- und Knieimplantaten, basierend auf der aktuellen Evidenzlage internationaler Endoprothesenregister und Metaanalysen?

**Material und Methode:**

Mittels des direkten Vergleichs von Daten aus den Endoprothesenregistern acht verschiedener Länder (USA, Deutschland, Australien, UK, Schweden, Norwegen, Neuseeland, Niederlande), dem Vergleich von 22 Review-Studien und Metaanalysen basierend auf Registerdaten, sowie der Auswertung der Empfehlung von Gesundheitssystemen unterschiedlicher Nationen wird eine Empfehlung generiert. Dazu wurden Reviews und Metaanalysen ausgewählt, deren Ergebnisse statistisch signifikant sind, ebenso wie die zum Zeitpunkt der Erstellung des Artikels aktuellen Jahresberichte der Endoprothesenregister.

**Ergebnisse:**

Für Knieendoprothesen kann eine lange Überlebensdauer sowie ein geringeres Revisionsrisiko mithilfe einer zementierten Verankerung mit antibiotikabeladenem Knochenzement erreicht werden. Bei Patienten ab einem Alter von 70 Jahren reduziert eine zementierte Verankerung des Hüftschaftimplantats das Risiko einer intra- oder postoperativen periprothetischen Fraktur um das Vierfache, dies gilt sowohl für elektive Hüfttotalendoprothesen (Hüft-TEP), als auch für Hemiendoprothesen nach Schenkelhalsfrakturen. Ein antibiotikabeladener Knochenzement reduziert signifikant (*p* = 0,041) das Risiko für das Auftreten einer periprothetischen Infektion, insbesondere bei Patienten mit Schenkelhalsfrakturen.

**Schlussfolgerungen:**

Die mit antibiotikabeladenem Knochenzement versorgte Knieendoprothese ist in Deutschland etabliert und wird durch die Evidenzlage bestätigt. Registerdaten und Metaanalysen empfehlen eine zementierte Verankerung des Hüftschaftimplantats beim älteren Patienten – hier sollte die Praxis in Deutschland der aktuellen Evidenz folgen.

## Abstract (en)

**Background:**

Hip and knee implants can be either fixated without cement, press-fit, or with bone cement. Real-world data from arthroplasty registers, as well as studies, provide a broad database for the discussion of cemented versus uncemented arthroplasty procedures.

**Objectives:**

What is the recommendation for cemented or cementless anchorage of hip and knee implants based on the current evidence from international arthroplasty registries and meta-analyses?

**Methods:**

A recommendation is generated by means of a direct comparison of data from the arthroplasty registries of eight different countries (USA, Germany, Australia, UK, Sweden, Norway, New Zealand, and The Netherlands), the comparison of 22 review studies and meta-analyses based on registry data, as well as the evaluation of the recommendation of healthcare systems of different nations. For this purpose, reviews and meta-analyses whose results were statistically significant were selected, as were the annual reports of the arthroplasty registries that were current at the time of writing.

**Results:**

For knee endoprostheses, a long survival time, as well as a lower risk of revision can be achieved with the help of cemented anchorage with antibiotic-laden bone cement. In patients aged 70 years and older, cemented anchorage of the hip stem implant significantly reduces the risk of intraoperative or postoperative periprosthetic fracture (times four), this applies both to elective total hip arthroplasties (TEPs) and to hemiarthroplasty after femoral neck fractures. Antibiotic-loaded bone cement significantly (*p* = 0.041) reduces the risk of periprosthetic infection, especially in patients with femoral neck fractures.

**Conclusions:**

Total knee replacement with antibiotic-loaded bone cement is well established in Germany and evidence based. Registry data and meta-analyses recommend cemented fixation of the hip stem in older patients—in Germany the evidence-based recommendations must still be transferred to daily practice.

Seit geraumer Zeit zeigt sich ein Trend zur zementfreien Versorgung von Hüft- und Knieimplantaten. Die zementierte Fixation wird mitunter als überholt angesehen. Welche Verankerungsmethode geht mit dem geringsten Revisionsrisiko und somit der längsten Überlebensdauer des Implantats einher? Dieser Frage soll im Folgenden mithilfe von Daten aus internationalen Endoprothesenregistern erörtert werden, um eine evidenzbasierte Entscheidung in der Praxis zu ermöglichen.

Arthrose gilt als der häufigste Grund für eine endoprothetische Versorgung insbesondere an Knie, Hüfte sowie Sprunggelenk und Fingern [26]. Das Gros der an Arthrose erkrankten Patienten ist älter als 60 Jahre, wobei Frauen deutlich häufiger betroffen sind als Männer. Schenkelhalsfrakturen (SHF) sind die zweithäufigste Ursache für eine endoprothetische Versorgung, insbesondere in der Patientenkohorte 80 Jahre und älter. Die Anzahl an intrakapsulären Schenkelhalsfrakturen hat zwischen 2009 und 2019 deutlich zugenommen mit einer Wachstumsrate von bis zu 23 % [53]. In der am häufigsten mit Hemiendoprothesen versorgten Altersgruppe von 80–89 Jahren liegt die jährliche Inzidenz von Schenkelhalsfrakturen bei 884 für Frauen und 569 für Männer pro 100.000 Einwohner [55]. Basierend auf der gestiegenen Anzahl von Arthrose- und Schenkelhalsfrakturpatienten steigerte sich auch die Zahl an Hüfteingriffen: innerhalb der OECD-Staaten ist Deutschland der Spitzenreiter mit 315 Hüftimplantationen pro 100.000 Einwohner im Jahr 2019 gefolgt von der Schweiz (313) und Österreich (295), wobei der Median der OECD-35-Staaten bei 174 Hüftimplantationen pro 100.000 Einwohner liegt [29, 47]. Im Vergleich dazu gibt es deutlich weniger endoprothetische Eingriffe am Knie: im Jahr 2019 wurden in Deutschland 227 durchgeführt. Hier liegen sowohl die Schweiz mit 260 Knieversorgungen pro 100.000 Einwohner, Finnland (242) und Österreich (229) vor Deutschland, während der Durchschnitt der OECD-33-Staaten bei 137 Knieimplantationen pro 100.000 Einwohner liegt [29, 47]. Die Knie- und Hüftendoprothetik ist somit ein häufiger Eingriff, wobei die Reduktion möglicher Revisionsrisiken immer das Ziel sein sollte, denn eine erhöhte Anzahl an Primäreingriffen birgt selbstverständlich auch immer das Risiko für eine steigende Anzahl an Revisionsprozeduren, welche mit hohen Kosten für die Kliniken und Krankenkassen, und möglichen Komplikationen für die Patienten einhergehen. Ebenfalls werden zunehmend ältere Patienten und Patienten mit multiplen Komorbiditäten (ASA III/IV) versorgt. Diese Faktoren begünstigen ein erhöhtes Revisionsrisiko. Nichtsdestotrotz hat sich aufgrund des technischen Fortschritts die Überlebensdauer der Implantate deutlich verlängert.

Nach 15 Jahren sind noch 93 % der Knie-TEP intakt

Laut einer 2019 im *Lancet* veröffentlichten Studie sind die Langzeitüberlebensraten für Knietotalendoprothesen (Knie-TEP) überzeugend: nach 15 Jahren sind noch 93 % der Knie-TEP intakt, nach 20 Jahren noch 90 % und nach 25 Jahren noch 82 % [19]. Die Überlebensraten für Hüfttotalendoprothesen (Hüft-TEP) sind mit 89 % nach 15 Jahren, 70 % nach 20 Jahren und 58 % nach 25 Jahren deutlich geringer [18].

## Aseptische Lockerung ist häufigster Revisionsgrund

Der aktuell häufigste Grund für die Revision einer Knie-TEP, wie im Endoprothesenregister Deutschland (EPRD) verzeichnet [17], ist eine aseptische Lockerung (23,5 %) gefolgt von Infektionen (15 %). Revisionseingriffe werden mit hohen finanziellen Belastungen für die Kliniken assoziiert, ebenso mit negativen gesundheitlichen Folgen für den Patienten. In der Regel erfordern mehr als die Hälfte aller Revisionen auch eine vollständige Re-Implantation aller primären Implantatkomponenten. Auch bei Hüft-TEP sind die häufigsten Gründe für eine Revision eine aseptische Lockerung (24,4 %) sowie eine Infektion (16,7 %). Periprothetische Frakturen sind der dritthäufigste Grund (14,3 %) für die Revision einer Hüft-TEP [17]. Als Hauptgrund für eine frühzeitige Revision, innerhalb der ersten 2 Jahre, gelten Infektionen mit einem Anteil von mehr als 50 %. Nach einer Revision aufgrund einer periprothetischen Infektion (PPI) liegt die Wahrscheinlichkeit für eine Re-Revision bei 36 % und stellt damit ein erhebliches Risiko für den Patienten dar [17].

## Zementierte Knie-TEP als Goldstandard

Die zementierte Versorgung in der primären Knieendoprothetik dominiert in Deutschland: 95,2 % der primären Knie-TEP sind zementiert und weitere 3,5 % werden hybrid versorgt ([17]; Tab. 1). Selbst bei primären unikondylären Knieversorgungen sind 90,9 % zumindest einseitig zementiert.

**Table 1:** 

	EPRD	AOANJRR	NJR	LROI	SAR	AJRR
Register	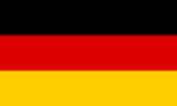	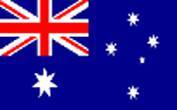	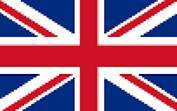	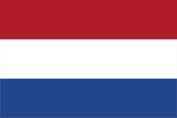	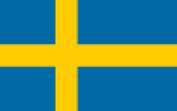	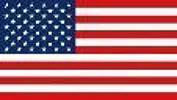
Zementiert	95,2 %	62,5 %	97,6 %^a^	92,6 %	90,5 %	79,0 %
Hybrid	3,5 %	18,6 %	0,2 %^a^	2,7 %	0,4 %	2,2 %
Zementfrei	1,2 %	18,9 %	2,1 %^a^	4,7 %	9,1 %	18,8 %

*AJRR* American Joint Replacement Registry, *AOANJRR* Australian Orthopaedic Association National Joint Replacement Registry, *EPRD* Endoprothesenregister Deutschland, *LROI* Landelijke Registratie Orthopedische Interventies, *NJR* National Joint Registry, *SAR *Swedish Arthroplasty Register

^a^Zur besseren Vergleichbarkeit wurden die unikondylären Schlittenprothesen herausgerechnet

Im internationalen Vergleich weisen das niederländische Endoprothesenregister (LROI) [15] und das schwedische Register (SAR) [57] vergleichbare Zementationsraten von über 90 % auf und auch im National Joint Registry (NJR) aus Großbritannien [42] dominiert mit 97,6 % die zementierte Verankerung der Knie-TEP. Über die letzten Jahre hinweg ist ein leichter Trend zur zementfreien Versorgung zu beobachten (Tab. 1). Die zementierte Versorgung dominiert auch im australischen Endoprothesenregister (AOANJRR) [4] mit einem Anteil von 62,5 %, allerdings ist gleichzeitig der Anteil an zementfreien Implantaten seit 2018 deutlich gestiegen und hat 2021 bereits einen Anteil von 18,9 % erreicht. Dieser Trend folgt den Entwicklungen aus den USA, die für das Jahr 2021 im American Joint Replacement Registry (AJRR) [1] einen Anteil von 18,8 % an zementfreien Knie-TEP ausweisen (Tab. 1). Dieser Trend wird gestützt durch neue Implantatentwicklungen, die als „modern“ im Markt angepriesen werden. Auch der Einsatz von Robotik kann als Tendenz zur vermehrten Verwendung zementfreier Implantate interpretiert werden. Ähnlich wie bei der Entwicklung des „Robo-Docs“ werden bei robotergestützten Operationen neben zementierten auch zementfreie Implantate verwendet [38].

Die Entscheidung für eine Fixationsmethode sollte im Idealfall auf wissenschaftlichen Erkenntnissen basieren, sowie auf Überlebensraten und Ausfallwahrscheinlichkeiten. Genau diese Evidenz sollen die Endoprothesenregister zur Verfügung stellen: sie wurden ursprünglich in Skandinavien eingerichtet, um klinisch relevante Informationen aus gebündelten Daten zu erhalten, die Versorgungsqualität zu verbessern und die Zahl der Revisionseingriffe zu verringern. Somit stellen Endoprothesenregister einen guten Überblick über verlässliche Implantatdesigns und Fixationsmethoden dar [32]. Für zementfreie Knie-TEP zeigt das EPRD eine deutlich erhöhte Ausfallwahrscheinlichkeit nach 5 Jahren (4,3 %) gegenüber einer zementierten oder hybriden Fixation (3,6 und 3,8 %) (Tab. 2).

**Table 2:** 

Ausfallwahrscheinlichkeiten innerhalb von …	1 Jahr	2 Jahren	3 Jahren	4 Jahren	5 Jahren	6 Jahren	7 Jahren
*Knietotalendoprothesen*							
Zementiert	1,7 [1,6; 1,7]	2,6 [2,5; 2,6]	3 [3,0; 3,1]	3,4 [3,3; 3,5]	3,6 [3,6; 3,7]	3,9 [3,8; 4,0]	4,2 [4,0; 4,3]
Hybrid	1,9 [1,7; 2,1]	2,8 [2,5; 3,0]	3,3 [3,0; 3,6]	3,5 [3,2; 3,8]	3,8 [3,5; 4,1]	4,1 [3,7; 4,5]	4,4 [3,9; 4,9]
Zementfrei	2 [1,6; 2,5]	3,2 [2,7; 3,9]	3,9 [3,3; 4,7]	4,1 [3,5; 4,9]	4,3 [3,6; 5,1]	4,6 [3,8; 5,5]	4,6 [3,8; 5,5]
*Elektive Hüfttotalendoprothesen, Schaftversorgung*							
Zementiert	2,3 [2,2; 2,4]	2,6 [2,5; 2,8]	2,9 [2,8; 3,0]	3,1 [3,0; 3,2]	3,3 [3,2; 3,5]	3,5 [3,3; 3,7]	3,8 [3,5; 4,0]
Zementfrei	2,7 [2,7; 2,8]	3,1 [3,1; 3,2]	3,4 [3,3; 3,5]	3,6 [3,5; 3,7]	3,8 [3,7; 3,8]	3,9 [3,8; 4,0]	4,1 [4,0; 4,2]

**Figure 1:**
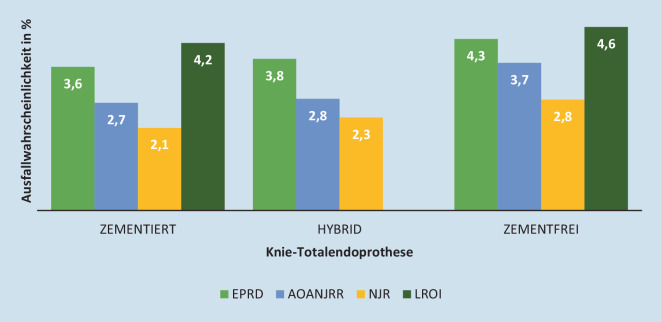

Vergleicht man diese Ergebnisse mit Daten anderer Register, kommt man zu demselben Ergebnis: die zementfreie Knieverankerung hat nach 5 Jahren eine um bis zu 37 % erhöhte Ausfallwahrscheinlichkeit im Vergleich zur zementfreien Verankerung (Abb. 1).

Zementierte Knieverankerung zeigt nach EPRD 2022 geringe Ausfallwahrscheinlichkeit

Dies wird auch durch einschlägige Literatur unterstützt: Ghandi et al. [22] belegten bereits 2009 die Überlegenheit der zementierten Knie-TEP durch höhere Überlebensraten gegenüber einer zementfreien Versorgung und damit einhergehend auch einem geringeren Revisionsrisiko für aseptische Lockerungen. Die Ausfallwahrscheinlichkeit (Odds Ratio, OR) lag bei zementfreien Knie-TEP bei 4,2 (95 % CI, 2,7; 6,5, *p* < 0,0001).

Irmola et al. haben 2021 [27], basierend auf Daten der Nordic Arthroplasty Association (265.877 Knie-TEP) ebenfalls die 10-Jahres-Überlebensrate von zementfreien, zementierten, hybriden und invers hybriden Knie-TEP untersucht, mit dem Ergebnis, dass die zementfreie Fixation mit einem erhöhten Revisionsrisiko (Hazard Ratio 1,3, 95 % CI, 1,1; 1,4) im Vergleich zur zementierten Fixation einhergeht. Selbst nach 15 Jahren zeigten in dieser Untersuchung zementierte Knie-TEP noch gute Überlebensraten im Gegensatz zu zementfreien Knie-TEP mit den meisten Ausfällen. Die kumulative 15-Jahres-Revisionsrate minimal stabilisierter Knie-TEP ist bei zementierter Verankerung niedriger als bei zementfreier Versorgung, wobei die hybride Fixation laut AOANJRR [4] die niedrigsten Revisionsraten aufweist. Das britische NJR [42] und das neuseeländische Endoprothesenregister (NZJR) [56] bestätigen diese Erkenntnis, insbesondere bei Patienten im Alter von 65–74 Jahren. Besonders bei Patienten über 75 Jahren, deren Knie-TEP zementiert verankert wurde, ist die Revisionsrate signifikant niedriger als bei zementfreier oder hybrider Verankerung (NJR 2019) [27]. Das reduzierte Revisionsrisiko, welches mit einer zementierten Fixation einhergeht, lässt sich möglicherweise auf den „verzeihenden“ Effekt des Knochenzements zurückführen: durch den Einsatz von Knochenzement können mögliche Defizite in der Platzierung und im Prothesendesign ausgeglichen werden [30]. Neben den Überlebensraten wird zunehmend auch die Zufriedenheit der Patienten mit ihrem Implantat berücksichtigt. Analysen des NZJR zeigen keine signifikanten Unterschiede in kurzzeitiger und langfristiger Patientenzufriedenheit zwischen den unterschiedlichen Versorgungen auf, allerdings weisen auch hier zementfreie Knie-TEP höhere Revisionsraten im Gegensatz zu vollständig zementierten Knie-TEP auf [45].

**Figure 2:**
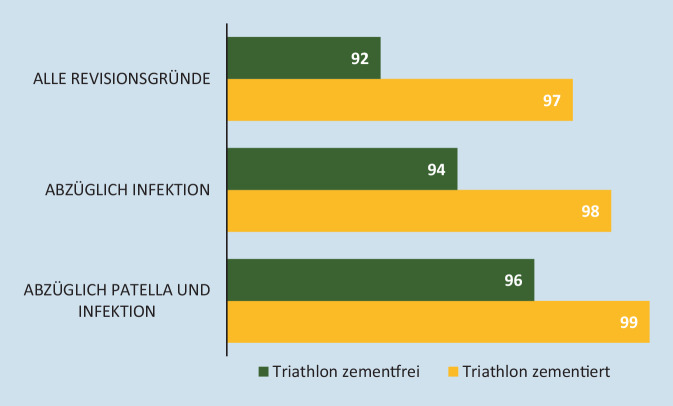

Der Einfluss des Prothesendesigns auf die Unterschiede in den Überlebensraten von Knie-TEP wird aktuell diskutiert. Das Design der Endoprothesen scheint sich durchaus auf die Standzeiten der Knieimplantate aus, dennoch zeigen Registerdaten, dass die zementierte Fixation im Vergleich zur zementfreien Knie-TEP mit geringeren Revisionsraten einhergeht. Eine Analyse des schwedischen Endoprothesenregisters (SAR) [57] zeigte, dass neben dem Prothesendesgin auch die Fixationsmethode einen Einfluss auf das Revisionsrisiko hat. Ein gleichzeitiger Anstieg des Anteils an zementfreien Knieprothesen (auf 10 %) und des Revisionsrisikos (auf 6 %) veranlassten die Autoren des SAR das Revisionsrisiko für die unterschiedlichen Fixationstypen zu analysieren. Anhand desselben Prothesendesigns (Triathlon-Prothese) wurden die Überlebensrate der zementierten und zementfreien Fixation miteinander verglichen (Abb. 2): die zementfreie Versorgung erreicht eine Überlebensrate von 92 % im Vergleich zur zementierten Fixation mit 97 % für alle Revisionsgründe. Selbst die Revisionsgründe abzüglich Patellaersatz und Infektion als Revisionsgrund zeigen, dass die zementfreie Verankerung mit 94 % respektive 96 % geringere Überlebensraten aufweist als die zementierte Verankerung mit 98 % bzw. 99 %. Neben der geringen Stabilität der zementfreien Knie-TEP könnte auch die Erfahrung des Orthopäden im Umgang mit dem zementfreien Implantat eine Rolle spielen [57].

Diese Theorie wird durch weitere Studien gestützt: neben der Wahl des Implantats und der Fixationsmethode hat die Erfahrung des Orthopäden einen entscheidenden Einfluss auf die Überlebensrate einer Knie-TEP [25]. Die Chirurgen, die mit ihrem Team für Knie-TEP lange Überlebensraten erzielten, entschieden sich mehrheitlich für die zementierte Verankerung in Kombination mit einer Endoprothese mit hochvernetztem Polyethylen und geringen Revisionsraten. Die Entscheidung des Orthopäden für oder gegen eine bestimmte Kombination aus Fixationsmethode und Implantat beeinflusst die Überlebensrate hierbei stärker als das Prothesendesign selbst [25].

Die aktuelle Evidenzlage bestätigt die zementierte (hybride) Knie-TEP als Goldstandard

Entgegen dem aufkommenden Trend der zementfreien Verankerung bestätigt die aktuelle Evidenzlage die zementierte (hybride) Knie-TEP als Goldstandard, mit dem geringsten Revisionsrisiko und den höchsten Überlebensraten.

## Paradoxon der zementfreien Hüftschäfte

Hüft-TEP werden in Deutschland zu 77 % zementfrei versorgt [17], auch im Vergleich zu anderen Ländern ergibt sich im Gegensatz zu Knie-TEP ein sehr heterogenes Bild (Tab. 3). Zur zementfreien Hüft-TEP tendieren auch die Niederlande (35 %) [15], sowie im besonderen Maße die USA (94 %) [1], wohingegen Schweden mit 67 % [57] und Großbritannien mit 62 % [42] einen deutlich höheren Anteil an hybriden bzw. zementierten Hüft-TEP aufweisen.

**Table 3:** 

	EPRD	AOANJRR	NJR	LROI	SAR	AJRR
Register	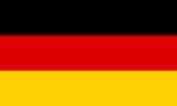	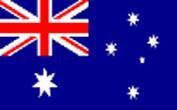	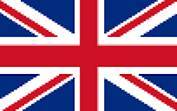	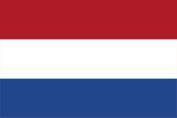	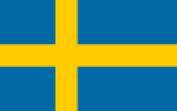	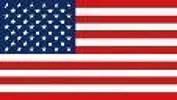
*Zementiert*	4 %	2 %	22 %	21 %	50 %	6 %
*Hybrid*	18 %	37 %	38 %	7 %	8 %	
*Hybrid, revers*	1 %	N/A	2 %	3 %	9 %	94 %
*Zementfrei*	77 %	61 %	35 %	69 %	33 %	

*AJRR* American Joint Replacement Registry, *AOANJRR* Australian Orthopaedic Association National Joint Replacement Registry, *EPRD* Endoprothesenregister Deutschland, *LROI* Landelijke Registratie Orthopedische Interventies, *NJR* National Joint Registry, *SAR* Swedish Arthroplasty Register

**Figure 3:**
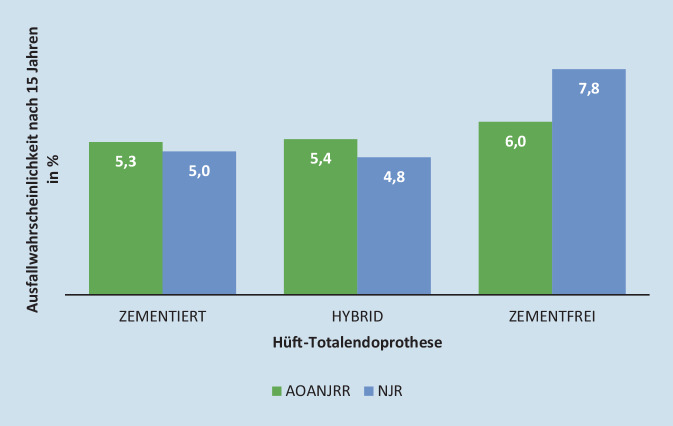

Wie unterscheidet sich ein zementfreies von einem zementierten Hüftschaftimplantat? Eine zementfreie Versorgung setzt voraus, dass das „press-fit“ eingebrachte Implantat zeitnah im Femur einwächst, was u. a. durch eine strukturierte Oberfläche der Prothese begünstigt werden kann. Nur gesundes und teilungsaktives Knochengewebe ist in der Lage, das Implantat vollständig zu umschließen und auf die Prothesenoberfläche zu wachsen. Ist der Patient von Osteopenie oder Osteoporose betroffen, ist die Teilungsaktivität des Knochengewebes erheblich beeinträchtigt und ein vollständiges Einwachsen des Implantats ist signifikant erschwert [2, 3]. In Europa sind schätzungsweise 22 % der weiblichen und 7 % der männlichen Bevölkerung ab einem Alter von 50 Jahren an Osteoporose erkrankt [31]. Laut EPRD-Jahresbericht wird die Patientengruppe der 75- bis 84-Jährigen am häufigsten mit elektiven Hüft-TEP versorgt. Somit wird die Patientengruppe, die häufig an den klinischen Folgen der Osteoporose leidet, hauptsächlich mit zementfreien Hüftschaftimplantaten versorgt. Das EPRD zeigt erhöhte Ausfallwahrscheinlichkeiten für zementfreie Hüft-TEP (3,8 %) nach 5 Jahren im Vergleich zur zementierten Verankerung (3,3 %) (Tab. 2), gleichzeitig belegen sowohl das AOANJRR als auch das NJR geringere Ausfallwahrscheinlichkeiten für zementierte (5,3 % bzw. 5,0 %) und hybride (5,4 % bzw. 4,8 %) Hüft-TEP nach 15 Jahren im Vergleich zu zementfreien (6,0 % bzw. 7,8 %) Hüft-TEP (Abb. 3). Einer der Hauptgründe für den Ausfall eines Hüftimplantats ist das Auftreten einer intra- oder postoperativen periprothetischen Fraktur. Sowohl die Revision aufgrund einer aseptischen Lockerung als auch bedingt durch eine periprothetische Fraktur, bergen für den älteren Patienten ein erhebliches gesundheitliches Risiko. Die Reduktion aller möglichen Revisionsrisiken zum Erhalt der Lebensqualität des älteren Patienten sollte angestrebt werden. Die Annahme, dass die Risiken einer zementfreien Versorgung mit einem erhöhten Revisionsrisiko für den älteren Patienten einhergehen, wird durch die Daten des AOANJRR [4] gestützt: ab einem Alter von 75 Jahren geht die zementfreie Hüft-TEP mit einem erhöhten Revisionsrisiko einher, wohingegen die zementierte Hüft-TEP das geringste Revisionsrisiko aufweist. Basierend auf dieser Erkenntnis empfehlen Babazadeh et al. [5] die zementierte Versorgung des Hüftschafts sowohl für erfahrene als auch unerfahrene Orthopäden.

Auch das EPRD hat in seinem Jahresbericht 2021 die Fragestellung „Schaftzementierung bei älteren Patienten ratsam?“ [16] beantwortet: ein zementierter Hüftschaft ist ratsam bei Patienten über 75 Jahren.

**Figure 4:**
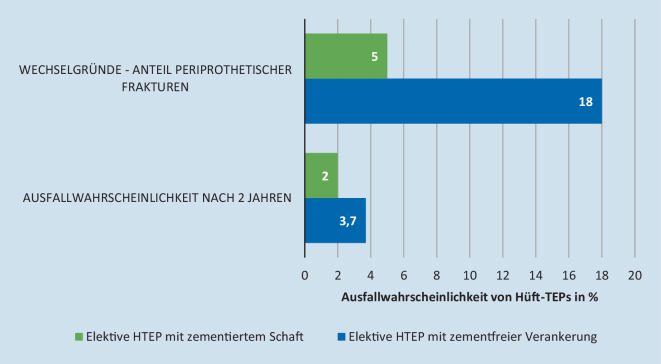

Bei Patienten ab einem Alter von 75 Jahren zeigte sich bei der zementierten Schaftversorgung eine deutlich geringere Ausfallwahrscheinlichkeit von 2,0 % (1,8 %; 2,2 %) im Gegensatz zur zementfreien Versorgung mit 3,7 % (3,4 %; 4,0 %) nachdem die Vergleichsgruppen statistisch angeglichen wurden. Der Anteil an periprothetischen Fakturen als Revisionsgrund war bei zementfreien Schäften mit 18 % deutlich erhöht gegenüber den zementierten Schäften mit nur 5 % (Abb. 4).

Eine zementfreie Versorgung erhöht das Risiko für periprothetische Frakturen

Empfehlungen zur Wahl der Fixationsmethode wurden von Bunyoz et al. [11] auf Basis einer Metaanalyse von 10 Endoprothesenregistern formuliert. Bei Patienten ab einem Alter von 75 Jahren sollte das Hüftschaftimplantat zementiert verankert werden, um das Revisionsrisiko zu senken und die Überlebensdauer der Hüft-TEP zu erhöhen! Diese Metaanalyse deckt sich mit der Analyse von 66.955 Hüftversorgungen des Norwegischen Endoprothesenregisters, 2021, [44] eine zementfreie Versorgung erhöht das Revisionsrisiko bedingt durch ein erhöhtes Risiko für periprothetische Frakturen und Dislokation.

Zementfreie Hüftschaftimplantate weisen 90 Tage nach der Operation ein 5fach erhöhtes Risiko (Relatives Risiko [RR] 5,2, CI 3,2; 8,5) für periprothetische Frakturen auf im Vergleich zu zementierten Schäften. Insbesondere für Frauen über 55 Jahre ist dies ein ernst zu nehmender Risikofaktor (RR 12, CI 6; 25) [12]. Dies betrifft allerdings auch junge Männer, wie eine Analyse des NZJR zeigte [46]: vermutlich bedingt durch ein unvollständiges Einwachsen des zementfreien Implantats in Kombination mit erhöhter physischer Aktivität zeigen zementfreie Hüftschaftimplantate bei jungen Männern vermehrt periprothetische Frakturen und Dislokationen. Als Umkehrschluss legt dies nahe, dass bei älteren Patienten, die eine, ggf. bedingt durch Osteoporose, schlechtere Knochenqualität aufweisen, ein Einwachsen des Implantats deutlich langsamer erfolgt und somit das Risiko für periprothetische Frakturen steigt. Auch Daten aus den USA, in denen zementfreie Hüft-TEP dominieren (94 %), zeigen, dass eine zementierte Verankerung des Hüftschaftimplantats mit einer statistisch signifikanten Reduktion der Revisionen aufgrund von periprothetischen Frakturen einhergeht (HR 0,113, 95 % CI, 0,052;0,245, *p* < 0,0001) [7]. US-Autoren um Springer et al. [54] untersuchten das Paradoxon der zementfreien Hüft-TEP in den USA und schlussfolgerten, dass ein zementierter Hüftschaft das Risiko für frühe periprothetische Frakturen reduziert und eine lange Standzeit (> 20 Jahre) ermöglicht. In Dänemark wurden über Jahre elektive Hüft-TEP primär zementfrei versorgt. Basierend auf der Evidenzlage zugunsten zementierter Hüft-TEP entschied sich Dänemark zu einer Änderung des Behandlungsalgorithmus: ältere Patienten, insbesondere Frauen, ab 60 Jahre sollen mit einem zementierten Hüftschaftimplantat versorgt werden. Im Nachgang analysierten Omari et al. [48] die Effekte des veränderten Behandlungsalgorithmus und dokumentierten eine signifikante Verringerung von periprothetischen Frakturen (von 4,57 % auf 1,25 %, *p* = 0,007) aufgrund der zementierten Fixation des Femurimplantats bei Frauen über 60 Jahren.

## Versorgung bei Schenkelhalsfrakturen

Analog zu den elektiven Hüft-TEP stellt sich auch im Fall einer Schenkelhalsfraktur die Frage: zementfreie oder zementierte Verankerung der Femurkomponente? Die multizentrische randomisierte kontrollierte Studie „World Hip Trauma Evaluation 5“ (WHiTE 5) analysierte neben dem Revisionsrisiko auch die wirtschaftlichen Aspekte einer zementierten bzw. zementfreien Versorgung nach dislozierten Schenkelhalsfrakturen [50]. Dabei wurden neben den tatsächlichen direkten Kosten für die Operation auch die durchschnittlichen Kosten für eine Nachbetreuung der Patienten über einen Zeitraum von 12 Monaten berücksichtigt. Zementierte Hüftschaftimplantate zeigten im Vergleich zu zementfreien Implantaten eine Kosteneinsparungen (961 Pfund) bei gleichzeitig gesteigerter Lebensqualität (QALY-Befragung der Patienten). Die Autoren kamen daher zu dem Schluss, dass die zementierte Hüft-TEP im Fall einer dislozierten intrakapsulären Schenkelhalsfraktur somit eine wirkungsvolle und gleichzeitig kosteneffiziente Behandlung ist [50].

Auch die Deutsche Gesellschaft für Orthopädie und Unfallchirurgie (DGOU) empfiehlt mit dem *Weißbuch Alterstraumatologie und Orthogeriatrie* [37] die zementierte Verankerung für Patienten mit Schenkelhalsfraktur beziehungsweise einer osteoporotischen Knochenstruktur. Die Zementierung der Femurkomponente ermöglicht eine bessere Verteilung der Kraft in den Knochen und reduziert dadurch das Risiko für weitere periprothetische Frakturen. Bei einem direkten Vergleich von Patienten, die nach einer SHF zementiert (610 Patienten) bzw. zementfrei (615 Patienten) versorgt wurden, traten in der zementfrei versorgten Patientenkohorte in 2,1 % der Fälle eine erneute periprothetische Fraktur auf, während 0,5 % in der zementiert versorgten Patientengruppe auftraten. Fernandez et al. (2022) zeigten, dass die zementierte Hemiendoprothese nach einer Schenkelhalsfraktur in einem geringeren Risiko für periprothetische Frakturen (0,5 % zementierte im Vergleich zu 2,1 % zementfrei) und einer signifikant besseren Lebensqualität für den Patient über 60 Jahre resultiert [21].

## Kosteneffiziente Versorgung

Da unser Gesundheitssystem unter Kostendruck steht, sollten Behandlungen langfristig kosteneffizient sein. Um die Ausgaben des britische National Health Service bei gleichbleibender Lebensqualität für den Patienten gering zu halten, wird eine evidenzbasierte Behandlungsempfehlung GIRFT („getting it right first time in orthopaedics“) für Hüftendoprothesen ausgesprochen: Patienten ab dem Alter von 70 Jahren sollen eine vollständig zementierte oder hybride Hüftendoprothese erhalten. Zur Reduktion der Ausgaben (2 Mio. AUS $ über 5 Jahre) des Australischen Gesundheitssystems empfehlen Blythe et al. [8] einen Wechsel des Behandlungsalgorithmus von einer zementfreien zu einer zementierten Versorgung sowohl bei elektiven Hüft-TEP als auch bei Hemiendoprothesen nach SHF. Ein umfassender Review aktueller Studien zur Kosteneffizienz unterschiedlicher Fixationsmethoden zeigt, dass eine zementierte oder hybride Verankerung die kosteneffizienteste Wahl ist. Ausschließlich in der sehr jungen Patientengruppe (unter 43 Jahren) stellte ein zementfreies Hüftschaftimplantat die kostengünstigste Option dar [58].

## Implantatdesign mit geringem Revisionsrisiko

**Figure 5:**
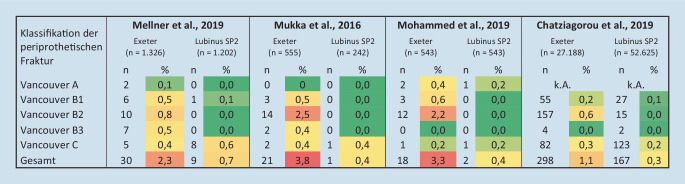

Die Evidenz empfiehlt eine zementierte Verankerung des Hüftschaftimplantats – aber mit welchem Implantatdesign? Ist ein poliertes Implantat ohne Kragen mit verjüngtem Schaft (z. B. Exeter, Stryker, Kalamazoo, MI, USA) oder ein anatomisch geformtes Implantat (z. B. Lubinus SPII, Waldemar Link, Hamburg, Deutschland) die beste Wahl (Abb. 5)? Die Implantatdesigns unterscheiden sich hinsichtlich dem Frakturrisiko: ein poliertes Implantat mit Kragen weist ein höheres Risiko für eine frühe periprothetische Fraktur im Vergleich zum anatomisch geformten Schaft auf [10]. Chatziagorou et al. [23] zeigten, basierend auf Daten des schwedischen Registers, den Einfluss des Implantatdesigns auf den Frakturtyp: das Risiko für Vancouver-Typ-B-Fraktur ist erhöht im Gegensatz zu Typ-C-Frakturen. Exeter-Schäfte weisen ein 10fach erhöhtes Risiko einer Typ-B-Fraktur auf verglichen mit Lubins-SPII-Schäften [23]. Dieser Zusammenhang ist in der Literatur mehrfach untersucht: zementierte, polierte, verjüngte Schäfte ohne Kragen zeigen eine erhöhte Inzidenz (3,8 %) von frühen periprothetischen Frakturen für Patienten über 80 Jahre im Vergleich zu zementierten, anatomischen Hüftschaftimplantaten (0,4 %) auf [41]. Mellner et al. [39] zeigen ebenfalls ein erhöhtes Risiko für periprothetische Frakturen für das Exeter-Implantat (2,3 %) im Vergleich zum Lubinus-SPII-Implantat (0,7 %) und empfehlen daher den Einsatz von Exeter-Implantaten bei SHF-Patienten mit größter Vorsicht zu betrachten. Ein polierter Schaft ohne Kragen, im Gegensatz zu einem anatomisch geformten Schaft, sollte laut Mohammed et al. [40] nicht in Patienten mit SHF oder einem Alter von über 80 Jahren eingesetzt werden, da diese eine erhöhte Inzidenz (3,3 %) für periprothetische Frakturen aufweisen. Die beste Versorgung von älteren Patienten und Patienten mit Schenkelhalsfrakturen bietet die zementierte Versorgung mit einem anatomisch geformten Hüftschaftimplantat mit Kragen.

## Keine erhöhte Mortalität durch Zementieren

Die zementierte Femurkomponente bietet viele Vorteile: Patienten sind sofort mobilisierbar, das Frakturrisiko ist gesenkt und die Gefahr des Einsinkens der Prothese ist reduziert. Dennoch entscheiden sich zahlreiche Orthopäden in der Praxis gegen eine zementierte Verankerung, vermutlich ausgelöst durch die Sorge vor möglichen intraoperativen Komplikationen. Fettembolien (auch als Knochenzementimplantationssyndrom bezeichnet oder Englisch „bone cement implantation syndrome“, kurz BCIS) kommen beim Zementieren häufiger vor als bei zementfreien Versorgungen. Beim BCIS wird ein kardiovaskulärer Kollaps ausgelöst. Dieser ist häufig assoziiert mit der Druckerhöhung im Knochen, der bei einer Druckbeaufschlagung des Knochenzements während des Zementiervorgangs, bei der Insertion der Prothese und beim Einschlagen des Implantats beobachtet werden kann. Eine Klassifizierung des Implantationssyndroms erfolgt in 4 unterschiedliche Abstufungen (Grad 0, 1, 2, 3) (Tab. 4). Das Risiko für einer Fettembolie kann jedoch signifikant reduziert werden durch konsequenten Einsatz der modernen Zementiertechnik unter Verwendung von Jet-Lavage und Maßnahmen zur Reduzierung des intramedullären Drucks [9, 33, 52].

**Table 4:** 

*Grad 0*	Kein Auftreten des Implantationssyndroms
*Grad 1*	Mäßige Hypoxie (arterielle Sauerstoffsättigung < 94 %) oder Hypotonie (ein Abfall des systolischen arteriellen Drucks > 20 %)
*Grad 2*	Schwere Hypoxie (arterielle Sauerstoffsättigung < 88 %) oder Hypotonie (ein Abfall des systolischen arteriellen Drucks > 40 %) oder unerwarteter Bewusstseinsverlust
*Grad 3*	Kardiovaskulärer Kollaps, der eine kardiopulmonale Wiederbelebung erfordert

Rassir et al. [52] bieten mit ihrer umfassenden Metaanalyse von 12 Studien einen guten Überblick über die Inzidenz des Implantationssyndroms: Grad 3 tritt nur in sehr seltenen Fällen mit einer Inzidenz von 0,1 % bei allen endoprothetischen Versorgungen auf und betrifft vor allem Patienten mit multiplen Vorerkrankungen (ASA III/IV). Das Syndrom kann bei endoprothetischen Eingriffen an allen Gelenken auftreten, mit der höchsten Inzidenz bei zementierten Hemiendoprothesen (0,4 % Grad 3) (Tab. 5). Die Analyse von 79.557 Patientendaten aus dem Norwegischen Endoprothesenregister zeigt keinen Unterschied im Mortalitätsrisiko für zementierte und zementfreie Hüft-TEP [13].

**Table 5:** 

	Ramsay et al. (2023) [51]	Bökeler et al. (2022) [9]	Rassir et al. (2021) [52]	Dale et al. (2020) [13]
*Anzahl Patienten*	*n* = 15.405	*n* = 92	*n* = 3294	*n* = 79.557
*Untersuchungszeitraum*	Bis zu einem Jahr nach Operation	Bis zu 3 Tage nach Operation	Bis zu 30 Tagen nach Operation	Bis zu 10 Jahren nach Operation
*ASA-Klassifikation Anteil Patienten in %*				
ASA 1	2 %	0 %	12 %	20 %
ASA 2	17 %	29 %	60 %	60 %
ASA 3	59 %	67 %	26 %	19 %
ASA 4	22 %	4 %	2 %	0 %
*Altersgruppe/Durchschnittsalter Patienten*	82 Jahre	84 Jahre	75 Jahre	< 65 Jahre: 36 % 65–74 Jahre: 35 % > 75 Jahre: 29 %
*Todesfälle 3 Tage nach Operation in %*	–	–	–	0,03 %
*Inzidenz BCIS Grad 3 in %*	1,70 %	0,00 %	0,40 %	N/A

*BCIS* „bone cement implantation syndrome“

Es gibt es keinen Beleg für eine erhöhte Mortalität bei zementierten Hüft-TEP

Die beobachteten Komplikationen, sowohl perioperativ als auch postoperativ standen im Zusammenhang mit dem Alter und den Komorbiditäten der Patienten – nicht mit dem Typ der Fixation. Die Inzidenz für die Entwicklung eines BCIS Grad 3 lag bei 0,03 %. Beim Vergleich von Daten des Australian Hip Fracture Registry mit dem Nationalen Todesindex [51] gibt es keine signifikante Assoziation zwischen der Verwendung von Knochenzement und der 30-Tages-Mortalitätsrate sowie ein Jahr nach dem Eingriff. Die zementierte Versorgung von SHF-Patienten ist sicher und erhöht nicht das Mortalitätsrisiko für den Patienten [51]. Bestätigt werden diese Erkenntnisse durch einen systematischen Review von Dominguez et al. [14]; hiernach gibt es keinen Beleg für eine erhöhte Mortalität bei zementierten Hüft-TEP oder Hemiendoprothesen nach SHF. Für jeden Patienten sollte also das erhöhte Risiko einer periprothetischen Fraktur bei unzementierter Versorgung gegen das überschaubare Risiko einer Fettembolie bei zementierter Verankerung abgewogen werden.

## Reduktion des Infektionsrisikos

Knochenzement kann auch als Wirkstoffträger für lokale Antibiotika dienen [33, 34]. Enthält der Knochenzement zur Verwendung bei primären Prozeduren nur ein Antibiotikum, so spricht man von einfach beladenem Knochenzement. Ein zweifach beladener Knochenzement enthält entsprechend zwei unterschiedliche Antibiotika und kann bei Revisionseingriffen oder der Versorgung von Hochrisikopatienten eingesetzt werden [24].

**Figure 6:**
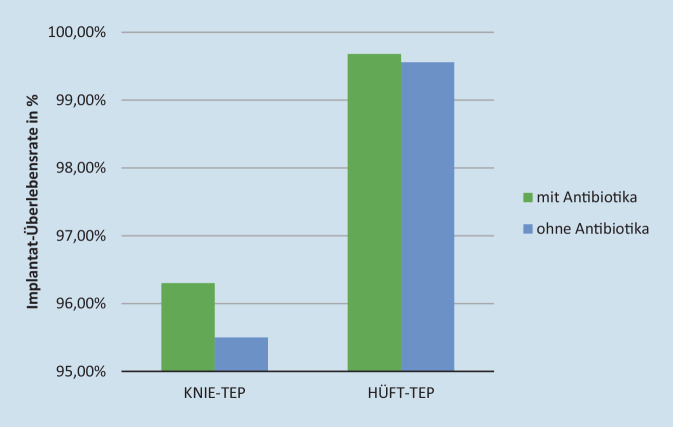

Eine umfassende Metaanalyse von Farhan-Alanie (370.000 Hüft- und 670.000 Knieendoprothesen) zeigt die schützende Wirkung von antibiotikabeladenem Knochenzement gegenüber einer PPI: die Ergebnisse sind statistisch signifikant für primäre Hüft-TEP (RR 0,6, 95 % CI 0,56–0,77; *p* < 0,001) [20]. In den USA wird der Einsatz von antibiotikabeladenem Knochenzement bei Primäreingriffen gerne angezweifelt, obwohl Parvizi et al. [49] bereits 2008 in einer umfangreichen Metastudie beweisen konnten, dass antibiotikabeladener Knochenzement das Infektionsrisiko bei Primäreingriffen signifikant senken kann. Eine weitere Untersuchung von 15.972 primären Knie-TEP an US-Veteranen (4741 Knie-TEP mit antibiotikafreiem Zement und 11.231 Knie-TEP mit antibiotikahaltigem Zement) zeigt niedrigere Revisionsraten aufgrund periprothetischer Infektionen in der Patientengruppe mit antibiotikabeladenem Knochenzement [6]. Jameson et al. [28] beobachten eine Implantatüberlebensrate der Knie-TEP mit antibiotikabeladenem Knochenzement nach 10 Jahren von 96,3 % im Vergleich zu 95,5 % mit antibiotikafreiem Zement (Abb. 6). Darüber hinaus wird bei Hüft-TEP mit antibiotikabeladenem im Vergleich zu antibiotikafreiem Knochenzement ein geringeres Risiko für PPI (HR 0,79) und gleichzeitige Reduktion des Revisionsrisikos bedingt durch aseptische Lockerungen oder Osteolysen aufgewiesen [36]. Kunutsor et al. [35] führten einen umfassenden Review zur Korrelation der Fixationsmethode (zementiert, zementfrei, hybrid, revers-hybrid) und dem PPI-Risiko primärer Hüft-TEP durch. Innerhalb der ersten 6 Monate zeigten die zementfrei versorgten Hüft-TEP ein erhöhtes PPI-Risiko im Vergleich zu allen zementierten Versorgungen (RR 0,75; 95 %; CI: 0,63–0,89). Im Gegensatz zu einer Verankerung mit antibiotikabeladenem Zement war die antibiotikafreie Versorgung mit einem erhöhten PPI-Risiko auch gegenüber den zementfreien TEP verknüpft [35]. Insbesondere SHF-Patienten mit multiplen Komorbiditäten weisen ein erhöhtes Risiko für das Auftreten einer periprothetischen Infektion auf. Die Verankerung von Hüftschaftimplantaten mit antibiotikabeladenem Knochenzement kann daher bei der Behandlung von Patienten mit Schenkelhalsfrakturen empfohlen werden [43].

## Fazit für die Praxis

- Zementierte Knietotalendoprothesen (Knie-TEP) zeigen, basierend auf internationalen Registerdaten, bessere Überlebensraten im Vergleich zu unzementierten Knie-TEP.
- Ein zementierter Hüftschaft ist laut Endoprothesenregister Deutschland ratsam bei älteren Patienten und geht einher mit einem geringeren Revisionsrisiko gegenüber unzementierter Versorgung.
- Die zementierte Hemiendoprothese nach einer Schenkelhalsfraktur resultiert in einem geringeren Risiko für periprothetische Frakturen und ist eine kosteneffiziente Versorgung.
- Registerdaten zeigen keine erhöhte Mortalität bei zementierten Hemiendoprothesen.
- Registerdaten und Metaanalysen belegen: antibiotikabeladener Knochenzement ist der Goldstandard in der Infektionsprophylaxe.

## Abkürzungen

AJRR: American Joint Replacement Registry
AOANJRR: Australian Orthopaedic Association National Joint Replacement Registry
ASA: American Society of Anesthesiologists
BCIS: „Bone cement implantation syndrome“
DGOU: Deutsche Gesellschaft für Orthopädie und Unfallchirurgie
EPRD: Endoprothesenregister Deutschland
GIRFT: „Getting it right first time in orthopaedics“
LROI: Landelijke Registratie Orthopedische Interventies
NJR: National Joint Registry
NZJR: New Zealand Joint Registry
OECD: Organisation for Economic Co-operation and Development
OR: Odds Ratio
PPI: Periprothetische Infektion
QALY: Quality Adjusted Life Years
RR: Relatives Risiko
SAR: Swedish Arthroplasty Register
SHF: Schenkelhalsfraktur
TEP: Totalendoprothese
WHiTE 5: World Hip Trauma Evaluation 5
